# Two-by-two factorial randomised study within a trial (SWAT) to evaluate strategies for follow-up in a randomised prevention trial

**DOI:** 10.1186/s13063-020-04373-4

**Published:** 2020-06-08

**Authors:** Lucy E. Bradshaw, Alan A. Montgomery, Hywel C. Williams, Joanne R. Chalmers, Rachel H. Haines

**Affiliations:** 1 Nottingham Clinical Trials Unit, University of Nottingham, Nottingham, NG7 2RD UK; 2 Centre of Evidence Based Dermatology, University of Nottingham, Nottingham, NG7 2NR UK

**Keywords:** Randomised, Retention, SWAT, Incentive, Short message service

## Abstract

**Background:**

Failure to collect outcome data in randomised trials can result in bias and loss of statistical power. Further evaluations of strategies to increase retention are required. We assessed the effectiveness of two strategies for retention in a randomised prevention trial using a two-by-two factorial randomised study within a trial (SWAT).

**Methods:**

Parents of babies included in the host trial were randomised to (1) short message service (SMS) notification prior to sending questionnaires at 3, 6, 12 and 18 months versus no SMS notification and (2) a £10 voucher sent with the invitation letter for the primary follow-up visit at 24 months or given at the visit. The two co-primary outcomes were collection of host trial (1) questionnaire data at interim follow-up times and (2) primary outcome at 24 months during a home/clinic visit with a research nurse.

**Results:**

Between November 2014 and November 2016, 1394 participants were randomised: 350 to no SMS + voucher at visit, 345 to SMS + voucher at visit, 352 to no SMS + voucher before visit and 347 to SMS + voucher before visit. Overall questionnaire data was collected at interim follow-up times for 75% in both the group allocated to the prior SMS notification and the group allocated to no SMS notification (odds ratio (OR) SMS versus none 1.02, 95% CI 0.83 to 1.25). Host trial primary outcome data was collected at a visit for 557 (80%) allocated to the voucher before the visit in the invitation letter and for 566 (81%) whose parents were allocated to receive the voucher at the visit (OR before versus at visit 0.89, 95% CI 0.69 to 1.17).

**Conclusion:**

There was no evidence of a difference in retention according to SMS notification or voucher timing. Future synthesis of SWAT results is required to be able to detect small but important incremental effects of retention strategies.

**Trial registration:**

ISRCTN registry, ID: ISRCTN21528841. Registered on 25 July 2014. SWAT Repository Store ID 25.

## Background

Failure to collect outcome data in randomised trials is inefficient, and can result in bias and loss of statistical power. A great deal of effort is put into recruiting participants to trials, but a focus on these recruitment targets may impact on retention [1]. In the United Kingdom (UK), Clinical Trials Unit directors identified ‘Methods to minimise attrition’ as one of the top three research priorities in a Delphi priority setting exercise in 2011/2012 [2]. However despite a large number of strategies being used for retention, there is a lack of evidence on which strategies are effective [3].

A Cochrane review in 2013 [4] reported on the effect of strategies to improve retention in randomised trials. Most of the 38 trials included were concerned with collection of outcome data via postal or electronic questionnaires rather than the return of participants to study sites. There was some evidence that monetary incentives for postal questionnaires and offer of monetary incentives for electronic questionnaires were effective in increasing response, but there were no trials that compared a monetary incentive given in advance with an incentive conditional on questionnaire completion. The review provided no evidence about the effectiveness of notifying participants using short messaging service (SMS, or text message) that a questionnaire had been, or was about to be, sent. The authors encouraged trialists to consider conducting adequately powered evaluations of retention strategies within their trials. Organisations such as Trial Forge have pointed out that even small incremental gains of 1%, when added together, can make a substantial difference to trial performance [5].

The BEEP randomised trial [6] investigated the effect of applying emollient for 12 months from birth on the development of eczema in high-risk infants. The primary outcome was diagnosis of eczema at 24 months in a face-to-face visit with a research nurse. Parents were also asked to complete electronic or postal questionnaires 3, 6, 12 and 18 months after randomisation. In a feasibility study for BEEP [7], data was unable to be collected for 13% of families at the final 6-month follow-up time point. The feasibility study employed frequent face-to-face and telephone contact with participating parents by research staff that was not planned for the full trial. Therefore, the main BEEP trial offered an opportunity to evaluate retention strategies in a trial with no face-to-face contact between randomisation and 24 months by conducting a randomised study within a trial [SWAT].

The aim of this SWAT was to compare the effectiveness of two strategies for participant retention on collection of outcome data in the BEEP trial.

## Methods

### Trial design

This was a two-by-two factorial randomised SWAT to evaluate two strategies to improve retention embedded within a randomised prevention trial. A brief protocol for the SWAT can be found on the SWAT repository store (SWAT ID 25) [8]. Participants in the SWAT were parents who had given consent for their infant to be randomised into the BEEP host trial. The SWAT was described in the participant information sheet for the host trial and, therefore, consent for the SWAT was implicit by consent for the host trial. Screening for eligibility and the consent process for the host trial was carried out during pregnancy or shortly after delivery. Randomisation took place at the same time for both the host trial and SWAT, and was within 21 days of the baby being born.

Follow up in the host trial was at 3, 6, 12 and 18 months by questionnaire and a visit at 24 months. A web-link to the questionnaires was sent in an email for parents to complete the questionnaires online. For parents that did not wish to complete questionnaires online, paper copies of the questionnaires were provided by post with pre-paid return envelopes. Where questionnaires were not completed or returned, a reminder was sent by email after 2 and 3 weeks of non-completion (or in the post for paper copies). The link to the online questionnaire remained active for 4 weeks after the initial email invitation was sent. Due to the lower than expected completion of questionnaires, the protocol was amended in May 2016 to allow members of the host trial team to telephone participants where questionnaires had not yet been completed but were still active. Text messages or emails were also sent by the trial team when they were unable to reach participants by telephone.

Host trial primary outcome data was collected during a face-to-face visit with a researcher at 24 months. If a face-to-face visit at 24 months was not possible then researchers attempted to collect the data via telephone, text, email or post. If contact could not be made the coordinating centre attempted to collect key minimal data, to be used in sensitivity analysis for the host trial, from the child’s general practitioner.

The following small gifts were sent to all parents by the coordinating centre:

- BEEP-branded muslin or bib at randomisation
- Birthday card and BEEP-branded plastic cutlery set or storybook at the child’s first birthday
- BEEP-branded cloth shoulder bag sent at 18 months

Parents were also sent trial newsletters every 6 months (from January 2016).

### Interventions

- *Intervention 1: Contact by short message service (SMS) versus no contact, prior to sending questionnaires at 3, 6, 12 and 18 months.*

For participants allocated to receive prior notification, a SMS message (text message) was sent the day before the email with the link to the questionnaire (or letter with the questionnaire) with the following wording:

- ‘Your BEEP study questionnaire will be ready soon. Please check your email tomorrow. Contact beep@nottingham.ac.uk if you have any problems completing it. Thanks!’
- For participants completing questionnaire on paper: ‘Your BEEP study questionnaire is on its way to you in the post. Contact beep@nottingham.ac.uk if you have any problems completing it. Thanks!’

- *Intervention 2: Compensation for parent’s time in the form of a £10 high-street shopping voucher sent to parents either before or given at the 24-month visit.*

At around 22 months, parents were sent a letter by the coordinating centre to ask them to get in touch with the research nurse to schedule the host trial 24-month visit. For participants allocated to be given the voucher before the visit, the letter included the following text:

*‘As a thank you for your ongoing participation with the study, we are enclosing in this letter a £10 voucher’.*

For participants allocated to be given the voucher at the visit, the letter included the following text:

*‘As a thank you for your ongoing participation with the study, your BEEP study nurse will give you a £10 voucher at the visit.’*

Research nurses rang parents to book appointments for the 24-month visit if they did not get in touch following the invitation letter.

### Randomisation

Research nurses randomised participants to the BEEP host trial by accessing an online system provided by the coordinating centre. A second randomisation was then automatically performed for the SWAT to each of the retention strategies (1:1) described above using an allocation schedule created by the Nottingham Clinical Trials Unit. SWAT allocation was stratified by recruiting site and host trial allocation and used a fixed block size of 4. Participants were informed in the host trial information sheet about the SWAT for SMS notification for questionnaires and timing of the voucher for the 24-month visit but were not informed at the time of their randomisation of their allocated groups for the SWAT.

The Trial Management Team and the research nurses were not blinded to the allocations for the SWAT. The sequence of allocations for the SWAT was concealed from the statisticians (LB and AAM) until the database was locked for the host trial.

### Outcomes

There were two co-primary outcomes:

1. Collection of data via the chosen method of questionnaire (electronic or postal) at host trial interim follow-up times (3, 6, 12 and 18 months)
2. Collection of the BEEP host trial primary outcome at 24 months during a home or clinic visit with a research nurse

Secondary outcomes were time to questionnaire completion and the number of reminders required to obtain questionnaire completion.

### Sample size

The sample size for the SWAT was determined by the sample size of the host trial. Based on the target of just under 1300 and assuming collection of outcome data from around 80–85% of participants (based on previous similar studies), a between-group absolute difference of ≥ 7 percentage points (equivalent odds ratio 1.7) could be detected with 90% power and 5% two-sided alpha.

### Statistical analysis

Analysis was conducted according to SWAT allocations for all randomised participants in the host trial, regardless of whether the allocated intervention was received. Between-group estimates are presented as odds ratios and absolute differences in percentage completion with 95% confidence intervals.

Collection of data via the chosen method of questionnaire was defined as completing either of the first two questions on the questionnaire. Collection of questionnaire data at interim follow-up times according to SWAT allocation for the prior SMS notification was analysed using Generalized Estimating Equations with the binomial family and logit link with an exchangeable correlation matrix to account for multiple observations per participant including allocated group in the host trial and questionnaire time point as covariates. An interaction term was also included between the intervention and questionnaire time point to explore whether there was any evidence that the intervention effect changed over time. The difference in the percentage of participants completing the questionnaire was also estimated with Generalized Estimating Equations using the binomial family and identity link. SWAT allocation for the timing of the voucher for the 24-month visit was not included in the models as this could not have had an effect on questionnaire completion as the questionnaire time points were prior to 24 months.

Collection of the host trial primary outcome at 24 months during a home or clinic visit with a research nurse for the two SWAT interventions was analysed using a multivariable logistic regression model including allocated group for the host trial as a covariate. The possibility of an interaction between the two SWAT interventions was investigated by the inclusion of an interaction term in the model. SWAT allocation for the prior SMS notification intervention was included in the model for completeness although was not expected to have an effect on the collection of host trial primary outcome data at 24 months.

Pre-planned sensitivity analyses for the co-primary outcomes were performed to explore: the extent of questionnaire completion according to allocated group for the prior SMS notification intervention, collection of host trial primary outcome via any method as well as the timing of the 24-month visit completion for both SWAT interventions and further adjustment for baseline variables with an observed imbalance between the groups (based on comparison of summary statistics only).

When the statistical analysis plan was written, it was thought that allocation to the SWAT was stratified by host trial allocation only. Therefore, the SWAT analysis plan specified that primary analyses would adjust for host trial allocation only. At the final analysis of the SWAT, it was discovered that trial site was also included as a stratification variable. Therefore, a sensitivity analysis using mixed-effects logistic regression was conducted to also include recruiting site as a random effect so that both variables used for stratifying allocation were included in the analysis.

Time to questionnaire completion was presented using Kaplan-Meier curves according to allocated group for the prior SMS notification intervention for each questionnaire time point. The effect of the prior SMS notification intervention on time to questionnaire completion was estimated using a Cox proportional hazards model including allocated group in the host trial as a covariate and using a shared frailty to account for the inclusion of four questionnaire time points for each participant. Questionnaires that were not completed were censored at 28 days. The number of reminders required to obtain questionnaire completion at each questionnaire time point was tabulated by allocated group for the prior SMS notification intervention along with the reasons that questionnaires were not completed.

#### Interim analysis

A separate interim analysis of the data for each of the two co-primary outcomes for the first 400 participants in the trial was planned to allow implementation of any strategy found to be superior for the remainder of the trial. Stopping boundaries for the interim analysis were calculated using the O’Brien and Fleming spending function for a total type-1 error of 0.05 for each co-primary outcome with one interim analysis. The interim analyses were conducted by statisticians at the coordinating centre independent of the trial and used the analysis methods described above.

Due to resource constraints, the interim analysis for the prior SMS notification for questionnaire completion was conducted later than planned and, therefore, included all questionnaires that had reached the end of the questionnaire completion window by the end of 2016. The stopping boundaries for the interim analysis for the prior SMS notification were, therefore, calculated based on 700 participants included in the interim analysis.

Full details of the analysis are documented in the statistical analysis plan for the SWAT, which was finalised prior to the first interim analysis (available on the host trial website https://www.nottingham.ac.uk/research/groups/cebd/projects/1eczema/beep-maintrial.aspx).

## Results

**Fig. 1:**
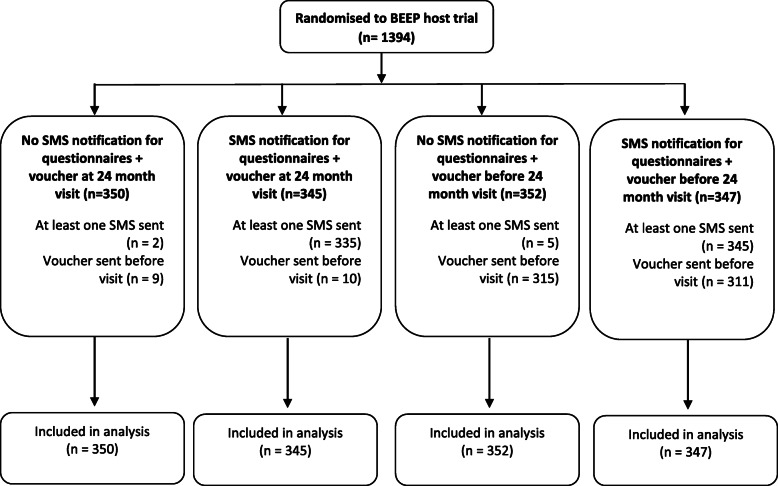
Participant flow diagram

Between November 2014 and November 2016, 1394 families were randomised to the host trial and SWAT (Fig. 1) at 12 hospitals and four general practice sites in the UK. The sample size was bigger than originally planned due to a planned sample size review by the Trial Steering Committee [6]. The interim analysis conducted in March 2017 for the prior SMS notification for questionnaires included 3239 observations from 1364 participants (713 participants who had reached the 12-month time point). The interim analysis for the timing of the £10 voucher for the 24-month visit included 403 participants and was conducted in December 2017. Neither of these analyses met the pre-specified stopping boundaries and, therefore, the SWAT continued as planned (Additional file 1). Follow-up for the host trial up to 24 months and SWAT was conducted between February 2015 and November 2018.

All randomised participants were included in the final analysis. Baseline characteristics were mostly balanced across the four groups, although there was a smaller proportion of families with three or more first-degree relatives with atopic disease in the group allocated to the prior SMS notification and the £10 voucher at the 24-month visit (Table 1).

**Table 1:** Baseline characteristics

	No SMS notification for questionnaires + voucher at 24-month visit (***n*** = 350)	SMS notification for questionnaires + voucher at 24-month visit (***n*** = 345)	No SMS notification for questionnaires + voucher before 24-month visit (***n*** = 352)	SMS notification for questionnaires + voucher before 24-month visit (***n*** = 347)
Host trial allocation				
Control	173 (49%)	175 (51%)	177 (50%)	176 (51%)
Intervention	177 (51%)	170 (49%)	175 (50%)	171 (49%)
Number of first-degree relatives with atopic disease^a^				
1	128 (37%)	125 (36%)	131 (37%)	123 (35%)
2	139 (40%)	167 (48%)	141 (40%)	149 (43%)
3 or more	83 (24%)	53 (15%)	80 (23%)	75 (22%)
Age of mother: mean [SD]	32.0 [5.0]	31.5 [5.3]	31.3 [5.3]	31.7 [5.4]
Number of other children in household				
0	133 (38%)	148 (43%)	146 (41%)	141 (41%)
1	150 (43%)	135 (39%)	141 (40%)	131 (38%)
2	49 (14%)	37 (11%)	53 (15%)	52 (15%)
3 or more	18 (5%)	25 (7%)	12 (3%)	23 (7%)
Decile of English Index of Multiple Deprivation 2015				
Median [25th, 75th centiles]	6 [4, 9]	6 [3, 9]	6 [3, 9]	5 [3, 8]
*n*	343	338	347	340

Data shown are *n* (%) using the number randomised to each group as the denominator unless otherwise specified

*SD* standard deviation, *SMS* short message service

^a^Host trial stratification variable

For the 692 participants allocated to receive prior SMS notification for the questionnaires, 680 were sent at least one SMS (98%). The majority of these participants were sent SMS notifications for three of the four questionnaire time points due to a server migration which meant that no SMS notifications were sent for a 4-month period (Table 2). The £10 voucher was sent with the letter asking the participant to contact the research nurse to arrange the visit for 90% of the 699 participants allocated to receive the voucher before the 24-month visit (Table 2). For 98 participants (14%) allocated to be given their voucher at the 24-month visit and 39 participants (6%) allocated to be given the voucher before the visit, it is not known whether they actually received the voucher (Table 2).

**Table 2:** Summary of short message service (SMS) notifications and timing of vouchers

	No SMS notification for questionnaires + voucher at 24-month visit (***n*** = 350)	SMS notification for questionnaires + voucher at 24-month visit (***n*** = 345)	No SMS notification for questionnaires + voucher before 24-month visit (***n*** = 352)	SMS notification for questionnaires + voucher before 24-month visit (***n*** = 347)
Number of SMS notifications sent^a^				
0	348 (99%)	10 (3%)	347 (99%)	2 (1%)
1	2 (1%)	1 (< 1%)	5 (1%)	3 (1%)
2	–	17 (5%)	–	17 (5%)
3	–	199 (58%)	–	204 (59%)
4	–	118 (34%)	–	121 (35%)
Voucher sent/given				
No	18 (5%)	10 (3%)	–	2 (1%)
Sent before 24-month visit	9 (3%)	10 (3%)	315 (89%)	311 (90%)
Sent/given on same day or after 24-month visit	254 (73%)	267 (77%)	9 (3%)	3 (1%)
Withdrew before 24 months	9 (3%)	13 (4%)	4 (1%)	16 (5%)
Voucher given – timing relative to visit not known	4 (1%)	3 (1%)	–	–
Not known^b^	56 (16%)	42 (12%)	24 (7%)	15 (4%)

Data shown are *n* (%) using the number randomised to each group as the denominator

^a^Note no SMS notifications were sent between 20 Oct 2016 and 15 Feb 2017

^b^Not known for 137 participants: 75 participants where the 24-month visit was not done and 62 participants where the 24-month visit was done

Overall questionnaire data was collected at interim follow-up times for 75% in both the group allocated to prior SMS notification and the group allocated to no SMS notification (Table 3). Between-group estimates for the effect of prior SMS notification versus none are presented for each time point (Table 3) as there was some evidence that the SMS intervention interacts with time (overall interaction *p* = 0.048). However, there was no evidence of a difference in collection of questionnaire data in the two groups at any time point. The sensitivity analysis conducted to explore the extent of questionnaire completion according to SMS notification allocation showed that completion of the key sections of the questionnaires were also very similar between the two groups as were the results from other sensitivity analyses (Additional file 1).

**Table 3:** Collection of questionnaire data at 3, 6, 12 and 18 months according to short message service (SMS) notification allocation

	No SMS notification for questionnaires (***n*** = 702)	SMS notification for questionnaires (***n*** = 692)	Adjusted difference in % collection (95% CI)	Adjusted odds ratio (95% CI)
**3 months**				
Collected	523 (75%)	535 (77%)	2.8% (−1.7 – 7.3%)	1.17 (0.91 – 1.49)
Not collected	179 (25%)	157 (23%)		
**6 months**				
Collected	528 (75%)	523 (76%)	0.4% (−4.1 – 4.9%)	1.02 (0.80 – 1.30)
Not collected	174 (25%)	169 (24%)		
**12 months**				
Collected	542 (77%)	516 (75%)	−2.6% (−7.1 – 1.8%)	0.87 (0.68 – 1.11)
Not collected	160 (23%)	176 (25%)		
**18 months**				
Collected	506 (72%)	503 (73%)	0.6% (−4.1 – 5.3%)	1.03 (0.82 – 1.30)
Not collected	196 (28%)	189 (27%)		

Data shown are *n* (%) using the number randomised to each group as the denominator

Estimates are adjusted for host trial allocation

5576 time points from 1394 participants included in model

Some evidence that SMS intervention interacts with time, becoming less effective at 6, 12 and 18 months, overall interaction *p* = 0.048; therefore, between-group estimates for the effect of prior SMS notification versus none are presented separately for each time point

If the interaction between the SMS intervention and time is ignored, the overall adjusted difference in data collection is 0.3% (95% CI −3.6 – 4.1%) and adjusted odds ratio is 1.02 (0.83 – 1.25)

Host trial primary outcome data at 24 months was collected at a face-to-face visit for 566 participants (81%) whose parents were allocated to receive the voucher at the visit and for 557 participants (80%) allocated to receive the voucher before the visit. Host trial primary outcome data collection was also similar according to SMS notification allocation (Table 4). There was no evidence of an interaction between the interventions for collection of the host trial primary outcome data (odds ratio for interaction 0.67, 95% confidence interval 0.39 to 1.14, *p* value 0.14); therefore, between-group estimates of the intervention effects are presented separately for each intervention. These showed no evidence of a difference in host trial primary outcome data collection at the visit according to SMS notification allocation or timing of the £10 voucher (Table 4). Collection of host trial primary outcome data via any method and timing of the 24-month visit were also similar according to allocated group as were the results from other sensitivity analyses (Additional file 1).

**Table 4:** Collection of host trial primary outcome data at 24 months during home or clinic visit by factorial margins

	No SMS notification for questionnaires (***n*** = 702)	SMS notification for questionnaires (***n*** = 692)	£10 voucher at 24-month visit (***n*** = 695)	£10 voucher before 24-month visit (***n*** = 699)
**Host trial primary outcome data collected during home or clinic visit**	558 (79%)	565 (82%)	566 (81%)	557 (80%)
Adjusted risk difference (95% CI)		2.4% (−1.8 – 6.5%)		−1.9% (−6.0 – 2.3%)
Adjusted odds ratio (95% CI)		1.15 (0.88 – 1.50)		0.89 (0.69 – 1.17)

Data shown are *n* (%) using the number randomised to each group as the denominator

Estimates are adjusted for host trial allocation

Odds ratio for interaction between study within a trial (SWAT) interventions from logistic regression model 0.67 (95% CI 0.39 – 1.14, *p* value 0.14)

**Fig. 2:**
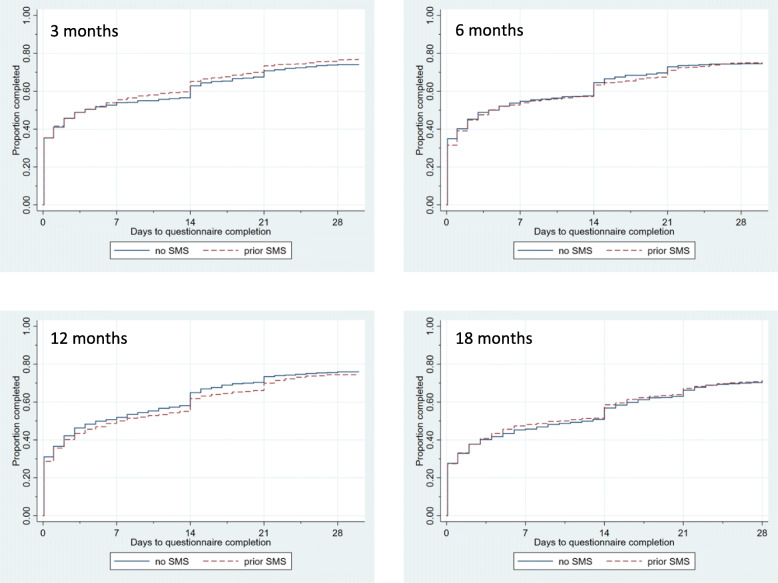
Kaplan-Meier curves for days to questionnaire completion according to short message service (SMS) notification allocation

The number of days to questionnaire completion from the date sent increased in both groups with each questionnaire follow-up time point; however, the time to completion was similar according to SMS notification allocation at all time points (Fig. 2, hazard ratio for time to questionnaire completion for SMS notification versus none 0.99, 95% confidence interval 0.87 to 1.13). Overall, between 50% and 60% of participants completed the questionnaire without a reminder at each time point. Similar percentages of participants in the two groups completed the questionnaire after the first reminder and second reminder at each time point (Additional file 1). The number of participants withdrawing consent from the host trial was small but was greater in the group allocated to receive the SMS notification at all time points. At 18 months, 27 participants (4%) allocated to SMS notification for questionnaires had withdrawn consent compared to 12 participants (2%) allocated to no SMS notification (Additional file 1).

## Discussion

In this SWAT, we did not find any evidence that either intervention improved completeness of host trial outcome data, or that SMS notification improved time to questionnaire completion or number of reminders required. Sensitivity analyses for questionnaire completion and host primary outcome data collection at the 24-month visit supported the main analyses.

A number of other randomised SWATs of SMS/text message notifications conducted in the UK since the Cochrane review have reported differing results. One trial observed an increased response to a postal questionnaire between 2 and 6 months after randomisation when an electronic prompt (SMS and/or email) was sent on the same day that the questionnaire was expected to be received compared to no electronic prompt (69% versus 61%) [9]. In another, SMS pre-notification did not increase response to a postal questionnaire at 3-month follow-up (83% versus 85% for no text) [10]. The results of the SWAT within the SUSPEND trial found a slight increase in response in the 4-week questionnaire in the SMS pre-notification group compared to no pre-notification (57% versus 52%) but no difference in response at 12 weeks (42% in both groups) [11].

There have also been two SWATs of timing of monetary incentives for trial retention since the Cochrane review. These have both been for postal questionnaire return, rather than trial visits, in the UK with either a gift voucher sent with the letter containing the questionnaire (unconditional incentive) or the letter stated that the voucher would be sent on completion of the questionnaire (conditional incentive). Hardy et al. found no evidence that an unconditional incentive of a £10 gift voucher increased response to a postal questionnaire at 1 year in a trial of a maternal intervention at child birth [12]. In contrast, Young et al. found that overall an unconditional incentive of a £5 gift voucher in a lung cancer screening trial in adults aged 50 to 75 years did increase response although the questionnaire return was very high (> 90%) in both groups at all time points (1, 3, 6 and 12 months) [13]. Since this SWAT started more SWATs of unconditional versus conditional trials of monetary incentives for retention have started [14], which are yet to report.

Our study has some limitations. Despite the relatively large sample size in BEEP, the confidence intervals for the estimate of the effects of the retention interventions are wide, meaning that we cannot rule out the possibility of small positive or detrimental effects that might be detected in a future meta-analysis of similar studies. The majority of participants in the SMS notification group only received text messages at three of the four time points due to a database error. The result for SMS intervention may have been different if the SMS notification was delivered as intended. In addition we do not know if the SMS notifications sent in this SWAT were received, errors may have been made in transcribing contact details and parents may have changed telephone numbers during the trial (although we did ask on each questionnaire and in the trial newsletters to let the coordinating centre know of any change in contact details). We also do not know if 137 participants were sent/given their vouchers including 62 participants where the 24-month visit took place due to the data not being entered on the database. This was only noted at the end of the trial, as central monitoring was not performed on data entry of the voucher number. We suspect that nurses forgot to enter this information as it was on a different system to the main trial database. A reconciliation of vouchers was undertaken at each site at the end of the trial, comparing the number of vouchers sent to the site, to the number expected to have been handed out, and the number remaining. This reconciliation showed that vouchers were given out as required; though many sites had some excess vouchers, owing to some families not wanting to accept the voucher. We also did not consider the cost of undertaking the SWAT study, including the resource implications at the coordinating centre for the different strategies. Future research may want to investigate the costs of the interventions. We did not seek any patient or public involvement in the design of this SWAT or the retention strategies investigated. This would be important to include for future SWATs, especially for SWATs addressing communication strategies as technology evolves. Inclusion of qualitative component in the SWAT to understand more about what the participants thought of the retention strategies may also have been useful to further understand the results.

An amount of £10 was chosen for the voucher at 24 months as this was similar to other studies and fitted in with the budget allocated within the trial for participant gifts. Higher value monetary incentives have been shown to increase response to postal questionnaires compared to lower value monetary incentives [4, 15] but it is not known if this is the same for completion of follow-up visits within a trial. We do not know if the vouchers given were used by the families; however, no vouchers were returned to the coordinating centre or research nurses.

The participant population in this SWAT was mainly mothers whose new babies were taking part in an eczema prevention trial with follow-up to 24 months after birth. Parents’ lives may be particularly busy during this time so other factors may have dominated who completed the questionnaires and attended the follow-up visits than the retention strategies investigated. Therefore, these results may not be generalisable to other populations. The intervention effects may also be different if the other small gifts and newsletters, used to try and keep parents engaged with the trial, were not sent out and these were the only retention strategies employed in the host trial.

A James Lind Alliance Priority Setting Partnership has recently agreed a list of the top 10 unanswered questions about participant retention in randomised trials to guide future methodological work [16]. ‘How could technology be best used in trial follow-up processes?’ featured at number 6. The use of incentives was one of the 21 questions taken to the face-to-face consensus meeting to agree the top 10 and was ranked at number 19 showing that the strategies investigated in this SWAT remain important to the UK clinical trial community. Further SWATs are required to answer the top 10 questions about trial retention. In the UK this is being encouraged by trial funders. The NIHR Health Technology Assessment Programme, for example, now allows and encourages trial applications to include a SWAT up to the value of £10,000 within the main application [17]. However, in order to minimise research waste, researchers should consider the five criteria suggested by Trial Forge [18] on whether a retention intervention needs further evaluation before conducting a SWAT.

## Conclusion

We did not find evidence that prior SMS notification was effective in increasing questionnaire data collection or for a voucher included in the invitation letter increasing host trial primary outcome data collection at a follow-up visit compared to the offer of a voucher. Using SMS notification as a reminder for questionnaire completion may be more appropriate than prior notification. Further research of the potential incremental effects of incentives for trial visit completion is required. It is critical that such SWATs are registered at the SWAT store to minimise duplication and to ensure that similar outcomes are used to facilitate meta-analysis to detect modest but important incremental effects.

## Supplementary information

**Additional file 1.** Sensitivity analyses, secondary outcomes and interim analyses.

## Data Availability

The datasets used and analysed during the current study are available from the corresponding author/Nottingham Clinical Trials Unit on reasonable request.

## Abbreviations

SMS: Short message service
SWAT: Study within a trial
