# Supplementary material for: Two-by-two factorial randomised study within a trial (SWAT) to evaluate strategies for follow-up in a randomised prevention trial
Source: Trials. 2020 Jun 8;21:529. doi: 10.1186/s13063-020-04373-4 (PMC7296963; doi:10.1186/s13063-020-04373-4)
Supplement: Supplementary file 1 — Additional file 1. Sensitivity analyses, secondary outcomes and interim analyses. [file 13063_2020_4373_MOESM1_ESM.docx]

**Additional file 1 – sensitivity analyses, secondary outcomes and interim analyses**

Sensitivity analysis for co-primary outcome 1 - Collection of data via the chosen method of questionnaire (electronic or postal) at host trial interim follow up times (3, 6, 12 and 18 months)

1. Additional adjustment for baseline variables with observed imbalance

*Table 1: odds ratios for collection of questionnaire data at 3, 6, 12 and 18 months for SMS notification v no SMS notification with additional adjustment for number of first degree relatives with atopic disease*

|  | **Odds ratio adjusted for host trial allocation and number of first degree relatives with atopic disease**  **(95% CI)** |
| --- | --- |
|  |  |
| **3 months** | 1.17 (0.92 to 1.50) |
|  |  |
| **6 months** | 1.03 (0.80 to 1.31) |
|  |  |
| **12 months** | 0.87 (0.68 to 1.11) |
|  |  |
| **18 months** | 1.04 (0.82 to 1.31) |
|  |  |
|  |  |
| ***Model ignoring interaction between SMS intervention and time*** | 1.02 (0.83 to 1.26) |
|  |  |

Data shown are n (%).

1. Repeating primary analysis using mixed effects logistic regression model to include recruiting site as random effect

*Table 2: Adjusted odds ratio for questionnaire data collection for SMS notification versus none*

|  | **Adjusted odds ratio**  **(95% CI)** |
| --- | --- |
|  |  |
| **3 months** | 1.49 (0.85 to 2.64) |
|  |  |
| **6 months** | 1.03 (0.59 to 1.81) |
|  |  |
| **12 months** | 0.65 (0.37 to 1.14) |
|  |  |
| **18 months** | 1.08 (0.63 to 1.85) |
|  |  |
| **p-value for interaction between SMS intervention and time** | p = 0.046 |
|  |  |
|  |  |
| *Model ignoring interaction between SMS intervention and time* | *1.02 (0.66 to 1.57)* |
|  |  |

Adjusted odds ratios estimated from model with fixed effects for host trial allocation and random effects for recruiting site and participant.

1. extent of questionnaire completion

*Table 3: Extent of questionnaire completion at 3, 6, 12 and 18 months by SMS notification versus none*

|  | **No SMS notification for questionnaires**  **(n = 702)** | **SMS notification for questionnaires**  **(n = 692)** |
| --- | --- | --- |
|  |  |  |
| **3 months** |  |  |
| Completed all key sections | 514 (73%) | 515 (74%) |
| Did not complete all key sections | 8 (1%) | 17 (2%) |
| None completed^1^ | 180 (26%) | 160 (23%) |
|  |  |  |
| **6 months** |  |  |
| Completed all key sections | 505 (72%) | 506 (73%) |
| Did not complete all key sections | 19 (3%) | 14 (2%) |
| None completed^1^ | 178 (25%) | 172 (25%) |
|  |  |  |
| **12 months** |  |  |
| Completed all key sections | 514 (73%) | 493 (71%) |
| Did not complete all key sections | 20 (3%) | 20 (3%) |
| None completed^1^ | 168 (24%) | 179 (26%) |
|  |  |  |
| **18 months** |  |  |
| Completed all key sections | 499 (71%) | 497 (72%) |
| Did not complete all key sections | - | - |
| None completed^1^ | 203 (29%) | 195 (28%) |
|  |  |  |

Data shown are n (%) using the number randomised to each group as the denominator.

Key sections of the questionnaire are shown in the table below

1 – None completed category includes participants who did not complete the questionnaire and participants who started the questionnaire but did not complete any of the key sections (4 participants at 3 months, 7 at 6 months, 11 at 12 months and 13 at 18 months).

| **3 month key sections** | **6 month key sections** | **12 month key sections** | **18 month key sections** |
| --- | --- | --- | --- |
| Diagnosis with eczema by a doctor or a nurse (secondary outcome) | Diagnosis with eczema by a doctor or a nurse  (secondary outcome) | Diagnosis with eczema by a doctor or a nurse  (secondary outcome) | Diagnosis with eczema by a doctor or a nurse  (secondary outcome) |
| Skin infections  (safety) | Skin infections  (safety) | Skin infections  (safety) |  |
| Emollient/moisturiser use  (compliance) | Emollient/moisturiser use | Emollient/moisturiser use |  |
| Slippages  (safety) | Slippages  (safety) | Slippages  (safety) |  |
|  |  | UK Working party criteria questions  (secondary outcome) |  |

Sensitivity analysis for co-primary outcome 2 - collection of host trial primary outcome data at 24 months during a home or clinic visit with a research nurse

1. Additional adjustment for baseline variables with observed imbalance

*Table 4: odds ratios for host trial primary outcome data collection at 24 months during home or clinic visit adjusted for number of first degree relatives with atopic disease*

|  | **Odds ratio adjusted for host trial allocation and number of first degree relatives with atopic disease**  **(95% CI)** |
| --- | --- |
|  |  |
| **SMS notification for questionnaires versus none** | 1.15 (95% CI 0.88 to 1.50) |
|  |  |
| **£10 voucher sent before 24 month visit versus £10 voucher at 24 month visit** | 0.89 (95% CI 0.69 to 1.17) |
|  |  |

1. Repeating primary analysis using mixed effects logistic regression model to include recruiting site as random effect

*Table 5: Adjusted odds ratio for host trial primary outcome data collection at 24 months during home or clinic visit*

|  | **Adjusted odds ratio**  **(95% CI)** |
| --- | --- |
|  |  |
| **SMS notification for questionnaires versus none** | 1.15 (0.88 to 1.51) |
|  |  |
| **£10 voucher sent before 24 month visit versus £10 voucher at 24 month visit** | 0.89 (0.68 to 1.17) |
|  |  |

Adjusted odds ratios estimated from model with fixed effects for host trial allocation and random effect for recruiting site.

1. Summary of host trial primary outcome data collection at 24 months

*Table 6: Summary of collection of host trial primary outcome data at 24 months by factorial margins*

|  | **No SMS notification for questionnaires**  **(n = 702)** | **SMS notification for questionnaires**  **(n = 692)** | **£10 voucher at 24 month visit**  **(n = 695)** | **£10 voucher before 24 month visit**  **(n = 699)** |
| --- | --- | --- | --- | --- |
|  |  |  |  |  |
| **Host trial primary outcome data collected** |  |  |  |  |
| No | 90 (13%) | 94 (14%) | 86 (12%) | 98 (14%) |
| Yes | 612 (87%) | 598 (86%) | 609 (88%) | 601 (86%) |
|  |  |  |  |  |
| ***Method of collection*** |  |  |  |  |
| Face to face | 558 (79%) | 565 (82%) | 566 (81%) | 557 (80%) |
| Telephone/email | 39 (6%) | 26 (4%) | 31 (4%) | 34 (5%) |
| Post | 15 (2%) | 7 (1%) | 12 (2%) | 10 (1%) |
|  |  |  |  |  |
| ***Months from birth to data collection*** |  |  |  |  |
| Median [25^th^, 75^th^ centile] | 24.2 [23.5, 25.3] | 24.2 [23.6, 25.2] | 24.3 [23.6, 25.2] | 24.2 [23.5, 25.2] |
| Min, max | 22.4, 36.0 | 19.1, 35.5 | 22.5, 36.0 | 19.1, 35.5 |
|  |  |  |  |  |
| Collected prior to 23 months | 7 (1%) | 7 (1%) | 4 (1%) | 10 (1%) |
| Collected between 23 and 26 months | 584 (83%) | 569 (82%) | 583 (84%) | 570 (82%) |
| Completed after 26 months | 21 (3%) | 22 (3%) | 22 (3%) | 21 (3%) |
|  |  |  |  |  |
| Completed outside preferred window of 21 to 30 months | 3 (<1%) | 8 (1%) | 4 (1%) | 7 (1%) |
|  |  |  |  |  |
| **Reason if host trial primary outcome data not collected** |  |  |  |  |
| Parent/main carer withdrawal of consent | 26 (4%) | 42 (6%) | 32 (5%) | 36 (5%) |
| No response | 62 (9%) | 51 (7%) | 52 (7%) | 61 (9%) |
| Other | 2 (<1%) | 1 (<1%) | 2 (<1%) | 1 (<1%) |
|  |  |  |  |  |

Data shown are n (%) using the number randomised to each group as the denominator.

Secondary outcome – number of reminders required for questionnaire completion

*Table 7: Number of reminders required to obtain questionnaire completion according to SMS notification allocation*

|  | **No SMS notification for questionnaires**  **(n = 702)** | **SMS notification for questionnaires**  **(n = 692)** |
| --- | --- | --- |
|  |  |  |
| **3 months** |  |  |
| Questionnaire completed with no reminder | 400 (57%) | 423 (61%) |
| Questionnaire completed after first reminder | 75 (11%) | 59 (9%) |
| Questionnaire completed between 1st & 2nd reminder after telephone call/text | 4 (1%) | 6 (1%) |
| Questionnaire completed after second reminder | 37 (5%) | 41 (6%) |
| Questionnaire completed after additional manual reminder by NCTU | 7 (1%) | 6 (1%) |
| Questionnaire not completed | 179 (25%) | 157 (23%) |
|  |  |  |
| *Completion method* |  |  |
| Online | 468 (67%) | 463 (67%) |
| Paper | 39 (6%) | 55 (8%) |
| Telephone | 16 (2%) | 17 (2%) |
|  |  |  |
| *Reason questionnaire not done* |  |  |
| Withdrawal of consent | 3 (<0.5%) | 13 (2%) |
| No response | 176 (25%) | 144 (21%) |
|  |  |  |
| **6 months** |  |  |
| Questionnaire completed with no reminder | 407 (58%) | 398 (58%) |
| Questionnaire completed after first reminder | 84 (12%) | 67 (10%) |
| Questionnaire completed between 1st & 2nd reminder after telephone call/text | 2 (<0.5%) | 3 (<0.5%) |
| Questionnaire completed after second reminder | 32 (5%) | 51 (7%) |
| Questionnaire completed after additional manual reminder by NCTU | 3 (<0.5%) | 4 (1%) |
| Questionnaire not completed | 174 (25%) | 169 (24%) |
|  |  |  |
| *Completion method* |  |  |
| Online | 478 (68%) | 463 (67%) |
| Paper | 42 (6%) | 48 (7%) |
| Telephone | 8 (1%) | 12 (2%) |
|  |  |  |
| *Reason questionnaire not done* |  |  |
| Withdrawal of consent | 7 (1%) | 20 (3%) |
| No response | 167 (24%) | 149 (22%) |
|  |  |  |
| **12 months** |  |  |
| Questionnaire completed with no reminder | 410 (58%) | 384 (55%) |
| Questionnaire completed after first reminder | 82 (12%) | 69 (10%) |
| Questionnaire completed between 1st & 2nd reminder after telephone call/text | 5 (1%) | 6 (1%) |
| Questionnaire completed after second reminder | 30 (4%) | 46 (7%) |
| Questionnaire completed after additional manual reminder by NCTU | 15 (2%) | 11 (2%) |
| Questionnaire not completed | 160 (23%) | 176 (25%) |
|  |  |  |
| *Completion method* |  |  |
| Online | 479 (68%) | 455 (66%) |
| Paper | 44 (6%) | 45 (7%) |
| Telephone | 19 (3%) | 16 (2%) |
|  |  |  |
| *Reason questionnaire not done* |  |  |
| Withdrawal of consent | 11 (2%) | 23 (3%) |
| No response | 149 (21%) | 153 (22%) |
|  |  |  |
| **18 months** |  |  |
| Questionnaire completed with no reminder | 366 (52%) | 360 (52%) |
| Questionnaire completed after first reminder | 81 (12%) | 79 (11%) |
| Questionnaire completed between 1st & 2nd reminder after telephone call/text | 1 (<0.5%) | 3 (<0.5%) |
| Questionnaire completed after second reminder | 40 (6%) | 37 (5%) |
| Questionnaire completed after additional manual reminder by NCTU | 18 (3%) | 24 (3%) |
| Questionnaire not completed | 196 (28%) | 189 (27%) |
|  |  |  |
| *Completion method* |  |  |
| Online | 456 (65%) | 445 (64%) |
| Paper | 42 (6%) | 46 (7%) |
| Telephone | 8 (1%) | 12 (2%) |
|  |  |  |
| *Reason questionnaire not done* |  |  |
| Withdrawal of consent | 12 (2%) | 27 (4%) |
| No response | 184 (26%) | 162 (23%) |
|  |  |  |

Data shown are n (%) using the number randomised to each group as the denominator.

Interim analysis results

Interim analysis 1 conducted in March 2017: SMS notification for collection of questionnaire data

*Table 8: Collection of questionnaire data at 3, 6 and 12 months by SMS notification allocation*

*(a) Including all questionnaire time points where completion window reached by the end of December 2016.*

|  | **No SMS notification for questionnaires** | **SMS notification for questionnaires** | **Adjusted difference in % collection**  **(95% CI)** | **Adjusted odds ratio**  **(95% CI)** |
| --- | --- | --- | --- | --- |
|  |  |  |  |  |
| **3 months** | n = 684 | n = 680 |  |  |
| Collected | 507 (74%) | 524 (77%) |  |  |
| Not collected | 177 (26%) | 156 (23%) |  |  |
|  |  |  |  |  |
| **6 months** | n = 583 | n = 579 |  |  |
| Collected | 435 (75%) | 440 (76%) |  |  |
| Not collected | 148 (25%) | 139 (24%) |  |  |
|  |  |  |  |  |
| **12 months** | n = 358 | n = 355 |  |  |
| Collected | 277 (77%) | 263 (74%) |  |  |
| Not collected | 81 (23%) | 92 (26%) | 1%  (-3% to 5%) | 1.08  (0.86 to 1.36) |
|  |  |  |  |  |

Data shown are n (%).

**3239** observations from **1364** participants included in model.

Test statistic for extra prior SMS intervention from model (using logit link): **z = 0.67**

The embedded trial of extra prior SMS notification for questionnaires will stop at the interim analysis if |z|≥ 2.9626.

*(b) Including only participants who had reached the 12 month questionnaire completion window by the end of December 2016 (descriptive only)*

|  | **No SMS notification for questionnaires N=358** | **SMS notification for questionnaires N=355** | **Adjusted difference in % collection**  **(95% CI)** | **Adjusted odds ratio**  **(95% CI)** |
| --- | --- | --- | --- | --- |
|  |  |  |  |  |
| **3 months** |  |  |  |  |
| Collected | 247 (69%) | 259 (73%) |  |  |
| Not collected | 11 (31%) | 96 (27%) |  |  |
|  |  |  |  |  |
| **6 months** |  |  |  |  |
| Collected | 264 (74%) | 257 (72%) |  |  |
| Not collected | 94 (26%) | 98 (28%) |  |  |
|  |  |  |  |  |
| **12 months** |  |  |  |  |
| Collected | 277 (77%) | 263 (74%) |  |  |
| Not collected | 81 (23%) | 92 (26%) | -0.7%  (-6.3% to 4.9%) | 1.01*  (0.75 to 1.34) |
|  |  |  |  |  |

Data shown are n (%).

2139 observations from 713 participants included in model.

* overall odds ratio presented in table; however some evidence that SMS intervention interacts with time, becoming less effective at 6 and 12 months. Interaction odds ratios: 0.77 (0.58 to 1.01) and 0.69 (0.52 to 0.91) for SMS*6 and SMS*12 months respectively, overall interaction p=0.028.

Interim analysis 2 conducted in December 2017: Timing of vouchers for host trial primary outcome data collection at 24 months

*Table 9 Collection of host trial primary outcome data for participants randomised up to and including 27 August 2015**

* The 400th participant was randomised to the host trial on the 27th August 2015. Three other participants were also randomised on the 27th August 2015, the interim analysis therefore included 403 participants.

1. *Summary of collection of host trial primary outcome data by factorial margins*

|  | **No SMS notification for questionnaires (N=205)** | **SMS notification for questionnaires**  **(N=198)** | **£10 voucher at 24 month visit (N=200)** | **£10 voucher before 24 month visit (N=203)** |
| --- | --- | --- | --- | --- |
| **Host trial primary outcome data collected** |  |  |  |  |
| No | 33 (16%) | 29 (15%) | 31 (16%) | 31 (15%) |
| Yes^1^ | 172 (84%) | 169 (85%) | 169 (85%) | 172 (85%) |
| Data shown are n (%)  Included primary outcome data collected via remote methods (e.g. telephone, email, etc). | | | | |

1. *Between group comparison of host trial primary outcome data collection*

|  | **Adjusted difference in % collection** | **Adjusted odds ratio** |
| --- | --- | --- |
| **SMS notification for questionnaires versus no SMS notification** | 1.8 (95% CI -5.2 to 8.8) | 1.12 (95% CI 0.65 to 1.92) |
| **£10 voucher sent before 24 month visit versus £10 voucher given at 24 month visit** | -0.2 (95% CI -7.2 to 6.8) | 1.02 (95% CI 0.59 to 1.75) |

Odds ratio for interaction between embedded trial interventions from logistic regression model 0.61 (95% CI 0.2 to 1.81, p-value 0.37).
Test statistic for timing of vouchers for the 24 month visit from logistic regression model: **0.06** *(assuming no interaction between interventions).*The embedded trial of timing of vouchers for the 24 month visit will stop at the interim analysis if the absolute value of the test statistic is greater than **3.9286**.
